# The Centre for Speech, Language and the Brain (CSLB) concept property norms

**DOI:** 10.3758/s13428-013-0420-4

**Published:** 2013-12-20

**Authors:** Barry J. Devereux, Lorraine K. Tyler, Jeroen Geertzen, Billi Randall

**Affiliations:** 1 Department of Psychology, University of Cambridge, Cambridge, CB2 3EB UK; 2 Department of Theoretical and Applied Linguistics, University of Cambridge, Cambridge, UK

**Keywords:** Concepts, Property norms, Semantic features, Semantic similarity

## Abstract

Theories of the representation and processing of concepts have been greatly enhanced by models based on information available in semantic property norms. This information relates both to the identity of the features produced in the norms and to their statistical properties. In this article, we introduce a new and large set of property norms that are designed to be a more flexible tool to meet the demands of many different disciplines interested in conceptual knowledge representation, from cognitive psychology to computational linguistics. As well as providing all features listed by 2 or more participants, we also show the considerable linguistic variation that underlies each normalized feature label and the number of participants who generated each variant. Our norms are highly comparable with the largest extant set (McRae, Cree, Seidenberg, & McNorgan, 2005) in terms of the number and distribution of features. In addition, we show how the norms give rise to a coherent category structure. We provide these norms in the hope that the greater detail available in the Centre for Speech, Language and the Brain norms should further promote the development of models of conceptual knowledge. The norms can be downloaded at www.csl.psychol.cam.ac.uk/propertynorms.

**Electronic supplementary material:**

The online version of this article (doi:10.3758/s13428-013-0420-4) contains supplementary material, which is available to authorized users.

## Introduction

Knowing the meaning of a concrete object, such as *sofa*, *cat*, or *cabbage*, entails knowing both its individual identity and how it can relate to other concepts by forming categories, such as *furniture*, *animals*, or *vegetables*. Throughout the history of cognitive psychology, many accounts of the organization of conceptual knowledge have been based upon the idea that concepts are distributed representations consisting of semantic primitives, or *features*, and that the overlap and differences in such feature-based representations can explain both the individuality of objects and their relationship to one another (Cree, McNorgan, & McRae, 2006; Hampton, 1979; McRae, Cree, Westmacott, & de Sa, 1999; McRae, de Sa, & Seidenberg, 1997; Moss, Tyler, & Jennings, 1997; Rosch, 1975; Smith & Medin, 1981; Taylor, Moss, & Tyler, 2007; Tyler & Moss, 2001). In order to test such accounts, it is necessary to have a model of the semantic feature information participants are likely to possess, and this has usually been estimated using semantic property norm data.

Researchers have used semantic property norms to explore many aspects of the semantic representation and processing of objects. The individual features generated by participants in property norming studies provide stimuli to test various claims about the representation of conceptual knowledge (Cree, McNorgan, & McRae, 2006; Cree & McRae, 2003; Grondin, Lupker, & McRae, 2009; McRae, Cree, Westmacott, & de Sa, 1999; Randall, Moss, Rodd, Greer, & Tyler, 2004; Taylor, Devereux, Acres, Randall, & Tyler, 2012; Tyler & Moss, 2001) and its breakdown in cases of acquired brain damage (Garrard, Lambon Ralph, Patterson, Pratt, & Hodges, 2005; Greer et al., 2001; McRae & Cree, 2002; Moss, Tyler, & Jennings, 1997; Moss, Tyler, Durrant-Peatfield, & Bunn, 1998; Moss, Tyler, & Devlin, 2002; Rogers et al., 2004; Tyler & Moss, 1997). In particular, theoretically motivated statistics that relate to features—their frequency of occurrence across concepts and the likelihood that pairs of features co-occur—have been proposed as fundamental organizing principles of cognitive models, allowing precise, quantitative claims about the architecture of the conceptual processing system to be explored through computational modeling (Cree et al., 2006; Mirman & Magnuson, 2008; O’Connor, Cree, & McRae, 2009; Randall et al., 2004; Rogers et al., 2004). Features from property norms are also important to our understanding of the neural underpinnings of conceptual representation and processing (Clarke, Taylor, Devereux, Randall, & Tyler, 2013; Tyler et al., 2013) and their distributional characteristics have been used in classification models to further this understanding (Chang, Mitchell, & Just, 2011). The use of featural information that has been generated by property norms has also been useful in understanding the time course of conceptual processing (Clarke et al., 2013; Sudre et al., 2012).

The most detailed and extensive set of norms made available to date is the set collected by McRae, Cree, Seidenberg, and McNorgan (2005). This set of norms consists of feature listings for 541 concrete objects and provides information on type of feature and feature production frequency for each concept. McRae et al. (2005) have also developed a systematic method for producing collated norm lists from the data provided by individual participants in norming studies, which has proved to be a useful methodological template for other researchers (e.g., Kremer & Baroni, 2011). For example, McRae et al.’s (2005) methodology takes into account that participants can produce features that may be conjunctions or disjunctions of smaller units of information (e.g., *has four wheels*; *is green or red*). Such composite features are divided into separate features during the normalization process (*has four wheels* and *has wheels*; *is green* and *is red*). McRae et al. (2005) also used a cutoff point for the production frequency of features, with a minimum of 5 (out of 30) participants having to produce a feature for it to be included in the final feature set for a given concept. While other norms also exist (240 nouns and 216 verbs, Vinson & Vigliocco, 2008; 64 nouns, Garrard, Lambon Ralph, Hodges, & Patterson, 2001; 193 nouns, Randall et al., 2004; 1,808 nouns, verbs, adjectives, and other parts of speech, Buchanan, Holmes, Teasley, & Hutchison, 2013), the McRae norms are the most widely used and have been considered a gold standard model of semantic feature representations of concepts (Baroni, Evert, & Lenci, 2008; Devereux, Pilkington, Poibeau, & Korhonen, 2009; Kelly, Devereux, & Korhonen, 2013).

The present article offers a new set of property norms, many of which overlap with the McRae et al. (2005) set, to add to the body of existing knowledge. The aim of this new set is to offer a more flexible tool that will enable an even wider use of property norms. Whereas the McRae norms contain features that have been produced by a minimum of 5 participants, our new set offers all the features that have been generated with a production frequency of two or more, enabling researchers to choose their own cutoff point and providing the base data that will help to establish which is the best cutoff point to capture shared knowledge while excluding idiosyncrasies associated with individual participants. In addition, we make available data on the linguistic variation in the features that the participants produced, by detailing which raw responses were mapped onto each normalized feature. These data include both syntactic variations (“does eat”; “is used for eating”; “is used in eating”; “is used to eat”) and the synonyms that have been incorporated into a single feature (“lives in the sea”; “lives in the ocean”; “is marine”). Our primary rationale for making available these linguistic variability data is that they will provide computational linguists with a useful resource for training and evaluating systems designed to automatically extract property-norm-like semantic feature representations from text corpora. In training such systems, the data on variations can be incorporated into the feature-learning framework to provide a bridge between the various wordings typically found in corpora and the normalized semantic feature labels. Furthermore, in evaluating such systems, human property norms are a useful “ground truth” that the model output can be compared against. A problem arises, however, because extracted features may be correct but not exactly match features in human norms, such as the McRae norms (e.g., the system may extract *lives in sea*, where *found in ocean* is the corresponding feature in the norms). In evaluating automatically acquired features against human data, researchers have therefore expanded a small number of the McRae feature labels by hand to include synonyms and inflectional variants (e.g., *lives on water* can be expanded to {*aquatic*, *lake*, *ocean*, *river*, *sea*, *water*}; see Baroni et al., 2008; Baroni & Lenci, 2009), although there is no guarantee that these are the variants people actually use. By providing the linguistic variability data for every feature, we aim to fill this gap.

Another innovative feature of our norms is that the data were collected online, so that participants were able to complete the experiment in their own homes. The data were then available in a digital format that permitted a significant degree of automated analysis. In particular, participants’ raw responses were processed using lexical rewrite rules, part-of-speech tagging from an automatic parser, and morphological decomposition. The purpose of this preprocessing was to automate and standardize as far as possible the steps required to map the variable raw feature responses to single semantic feature labels (e.g., the responses “is for a child” and “is for children” can be automatically mapped to the standardized feature *is for children*).

## Method

### Participants

One hundred twenty-three members of the Centre for Speech, Language and Brain (CSLB) subject pool, 18–40 years of age, right-handed, and native speakers of British English, took part in this study. Participants completed the study online, and the study was approved by the Cambridge Psychology Research Ethics Committee. Participants were able to take part in repeat sessions and completed between 1 and 11 sessions. Sessions were designed to last about an hour, with 30 concepts presented in each session. Participants received £6 in payment for each session.

### Stimuli

A total of 866 concrete concepts were selected for online norm completion. In selecting the concepts to be normed, we aimed to replicate the McRae norms as much as possible, and so we included all concepts from that set that were applicable to a British English environment (*n* = 490/541). We omitted concepts that are unfamiliar to Britons (e.g., *cougar, chickadee, caribou, tomahawk*). We selected additional items from the Snodgrass and Vanderwart (1980) pictures, from various other unnormed concrete concepts that we have used in previous studies, from items with high concreteness ratings (>550) in the MRC psycholinguistic database (Wilson, 1988), and from the category norms developed by Van Overschelde, Rawson, and Dunlosky (2004). One of the shortfalls of norms is the presence of unique and highly distinctive features that are not truly unique in the real world. Therefore, wherever possible, we tried to decrease the possibility of creating spurious unique properties by ensuring that all concepts had at least one other related concept in the list. For example, in the original McRae norms, the concept *dandelion* is the only flower and, therefore, has many unique features, including *is a flower*. We added other flowers (e.g., *buttercup, daisy, sunflower, pansy*) to accompany *dandelion*. Similar to McRae et al. (2005), we tried to avoid ambiguous concepts. Where the concept label was an ambiguous word, we provided a disambiguating term in parenthesis [e.g. “seal (animal)” and “organ (musical instrument)”]. There are 638 completed concepts in the current set, with data collection ongoing. A list of the concepts and their categories is included in the Appendix. These 638 concepts, their features, and feature variants constitute version 1 of the CSLB norms, available online at www.csl.psychol.cam.ac.uk/propertynorms. As additional concepts are completed, they will be incorporated into later versions of the norms, which will be made available on the Web site.

### Norm collection

Participants from the CSLB subject pool completed the norms anonymously online by responding to an e-mail invitation. This took them to the online norm Web page, where they gave consent to participate in the study. A screen of instructions then appeared. The method of providing features was described, and a short video showing examples of how to fill in the norms was shown. Once they had seen the video, the participants began the main part of the study.

**Fig. 1:**
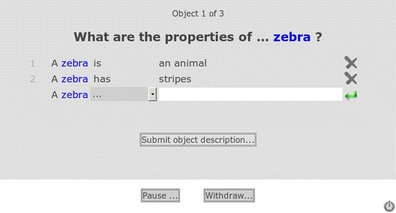
Screen shot of the norming Web page showing how the features were collected

Participants were presented with a concept word (e.g., *zebra)* and space to add their features (see Fig. 1). The participants were asked to add a relation word chosen from a drop down menu. The relation words were *is*, *has*, *does*, *made of*, and *“…”* (participants were instructed to use “…” when they wished to use any other relation). Participants were asked to complete the features in the space provided and to generate at least five features per concept. Concepts were pseudorandomly selected to avoid consecutive presentation of concepts from the same category (e.g., animals, clothing) and to ensure that each concept was presented to 30 participants. Once the participants had completed their session, their data were saved to a text file for further analysis. In line with ethical considerations, participants were given the opportunity to withdraw from the study at any time, including before they had completed the 30 concepts in a session.

### Data analysis

#### Within concept

As far as possible, we automated the process of compiling lists of normalized features for each concept. Data from the 30 participants for each concept were concatenated, giving a single long list. This list then underwent various stages of processing, all of which related to the features provided by the participants and left the relation words untouched. Preprocessing removed specified adverbs, such as *really* and *very,* following the procedure used by McRae et al. (2005). The aim of this stage was to increase the level of consistency across participants to allow as high a proportion of features to be automatically collated as possible. In additional preprocessing, the terms in the features were tagged using the part-of-speech tagger in RASP, an automatic parser (Briscoe, Carroll, & Watson, 2006) and morphologically decomposed using Morpha (Minnen, Carroll, & Pearce, 2001). Thus, individual words in the features were tagged for part-of-speech, and morphologically complex words (past tense words, plurals, etc.) were decomposed into a stem and affix format (wings → wing+s; sings → sing+s; attached → attach +ed; ran → run +ed). Finally, individual pieces of information were extracted by feature-splitting rules, automating methodology described by McRae et al. (2005). For example, a feature such as *has a long neck* was rewritten as *has a long neck* and *has a neck*. At all stages in the development and use of the preprocessing system, rules were checked manually and corrected if necessary, to guard against overgeneralized modification of the features.

The resulting preprocessed features were then automatically collapsed, with a record of which participant had provided input into which feature. Table 1 shows a subset of the features given for the concept *turtle.*

**Table 1:** A subset of the preprocessed features for the concept *turtle* (only a sample of uncollapsed features is shown)

PF	Relation	Feature	Participant list
23	has	a shell	p15 17 18 24 28 30 39 45 48 50 52 55 56 58 59 60 61 63 64 88 132 133 135
18	does	swim	p15 18 28 30 45 48 52 55 56 58 59 60 62 88 113 131 131 133
16	does	lay	p15 17 24 28 39 55 56 59 59 60 62 87 88 113 132 133
14	does	live	p18 24 30 45 45 52 52 52 55 59 60 62 64 133
10	is	a reptile	p18 45 50 56 58 60 64 113 132 134
10	is	an animal	p28 39 45 48 50 53 132 133 134 135
9	does	lay egg+s	p15 24 60 62 87 88 113 132 133
8	is	green	p24 30 45 48 55 59 60 134
7	is	slow	p24 28 48 50 57 61 64
5	has	four leg+s	p52 55 62 88 133
5	does	have	p62 87 87 87 87
5	does	swim in sea+s	p28 56 59 131 133
4	is	endanger+ed	p39 45 55 58
4	has	a tail	p39 63 64 88
4	has	flipper+s	p60 113 131 134
4	has	skin	p24 28 58 63
3	does	eat	p52 60 88
3	does	live in sea+s	p18 59 60
3	does	live in water	p24 52 62
3	does	move	p17 52 62
3	has	a beak	p18 24 45
2	does	crawl	p52 60
2	has	small head	p30 57
2	does	lay egg+s on the beach	p28 39
2	has	scaly skin	p24 58
2	does	travel	p60 133
2	does	look	p17 45
2	is	old	p57 61
2	has	head	p30 57
2	…	be+s endanger+ed	p24 87
2	does	have a hard shell	p62 87
2	has	scale+s	p18 52
2	is	shy	p15 52
2	is	graceful	p15 134
2	does	live a long time	p45 52
1	does	live in shell+s	p52
1	…	can live on land or sea	p87
1	has	a little tail	p24
1	is	cold-blooded	p132
1	is	not+ dangerous	p28
1	is	crawl+s	p57
1	has	tough skin	p63
1	is	big	p50
1	does	lay egg+s on beach+s	p55
1	does	move slowly on land and quick in the sea	p17
1	does	return	p17
1	does	look like tortoise+s	p17

Preprocessing maintains a record of which participants have provided input into which feature. *PF*, production frequency

This automatically generated output was then worked on by hand. Features that result from the automatic feature-splitting process but make no sense were removed—for example, in the *turtle* example, “does lay” (which, while literally true, is not a distinct component action of the original feature “does lay eggs”). Spelling mistakes were corrected, and features where the wrong relation word had been selected (e.g., “does in the sea”) were also corrected when the intention was clear (“is in the sea”). Then the process of extracting units of information (e.g., decomposing “lays eggs on the beach” into “lays eggs on the beach” and “lays eggs”) was completed, following the McRae et al. (2005) methodology for feature normalization. Further synonyms were also identified and collapsed (e.g., “does kill”; “is lethal” became *does kill*). Features were collapsed as synonyms when the number of overlapping participants was minimal. For example, “made of cloth,” “made of material,” and “made of fabric” have been collapsed into a single feature (*made of fabric_cloth_material*), because these feature labels were generated by nonoverlapping sets of participants, indicating that participants had the same semantic feature in mind but used different labels to verbalize it. Occasionally, a single participant would produce two synonyms, but this tended to be for the less familiar concepts, where it might be difficult to generate five or more features. The process of collapsing across features that had been generated by the participants continued until no further synonyms could be identified. After these stages, all the concepts were considered together for the final stage of normalization.

#### Between concepts

The final stage of data analysis attempted to ensure that features are consistent across concepts. This is helped by the three processes that are part of the automatic procedure: synonym mapping, morphological decomposition, and qualifier removal. Once the concepts have been collated, they are placed in a list and sorted by keyword. When there are variations in the expression of a feature, these are standardized. For example, the features “is used in archery” and “is used by archers” were collapsed together as *is used in archery*. The features “does live in packs” and “does live in groups” were collapsed to make *does live in groups*. In order to obtain a heuristic for identifying synonymous feature pairs such as these, we performed latent semantic analysis (LSA; Landauer, McNamara, Dennis, & Kintsch, 2007) on the production frequency data. LSA is more typically applied to corpus data—specifically, a frequency matrix where rows correspond to words and columns correspond to documents (or contexts) and where each element of the matrix gives the frequency with which a particular word occurs in a particular document. Singular value decomposition of the frequency matrix is used to express the semantics of words in a lower dimensional space. Distances in this space reflect the degree of relatedness between words; two words are close in this space if they tend to occur in similar documents. Importantly, a pair of words need not occur together in the same documents in order to be similar; for example, the words “human” and “user” might never occur in the same documents but will be similar if they tend to occur in similar kinds of documents (Landauer, Foltz, & Laham, 1998). We exploit this principle of LSA here. We perform singular value decomposition on the feature × concept production frequency matrix (with features as rows), to reduce the dimensionality of feature representations to 50. Similarity between pairs of features was calculated as the cosine between them, and we identified pairs of features that were highly similar but were not necessarily listed together in the same concepts (e.g., *is absorbent* and *does absorb*). In this way, we could identify pairs that should potentially be normalized to the same feature label.

## Results

We report here the results from 638 completed concepts. For all the results reported in this section, we have removed the taxonomic features (e.g., *is a bird; is furniture*). Taxonomic features refer to a superordinate category and are not normally regarded as true semantic features in studies of conceptual representation (Grondin et al., 2009; McRae et al., 2005; Pexman, Hargreaves, Siakaluk, Bodner, & Pope, 2008; Taylor et al., 2012). The files that we make available include the taxonomic features, and taxonomic features are tagged as being such.

### Comparison with the McRae norms

The work by McRae and his colleagues has provided the current gold standard in the production of property norms. It is therefore important to determine whether our norms have a high degree of comparability with the McRae norms (while providing additional information and flexibility, which we feel is the additional strength of our norms). We compared our norms with the McRae norms for a subset of measures reported in McRae et al. (2005). To ensure the best comparison, we used the same production frequency cutoff of five. There are currently 415 concepts in common between the CSLB norms and the McRae norms, which are the items analyzed here.

Table 2 shows that our participants generated, on average, 2.15 features per concept more than McRae’s participants. This was significant, *t*(414) = 11.70, *p* < .001. Our norms have significantly more shared features (i.e. features occurring in three or more concepts), *t*(414) = 18.96, *p* < .001, and fewer distinctive features (i.e. features occurring in 1 or 2 concepts), *t*(414) = −5.57, *p* < .001, than do the McRae norms. In line with this, the mean distinctiveness of concepts in the CSLB norms is lower than the McRae norms, *t*(414) = −14.03, *p* < .001. High scores on mean distinctiveness indicate a higher proportion of distinctive features.

**Table 2:** Means (and standard deviations; *SD*s) of number of features (NOF), number of shared (NOsF) and distinctive (NOdF) features, and mean distinctiveness (MeanD) for the new CSLB norms and for the McRae norms and their respective correlations across concepts

		NOF	NOsF	NOdF	MeanD
McRae	Mean	12.2	8.2	4.1	0.35
	*SD*	3.3	3	2.6	0.16
CSLB	Mean	14.4	11	3.4	0.26
	*SD*	3.3	3.2	2.4	0.13
Diff		2.2	2.8	-0.7	−0.1
Correlation		.35	.51	.5	.63

Despite the differences between the two sets, they are highly correlated (all *p*s < .001; for Pearson correlation values, see Table 2), indicating that those concepts that generated many features in the McRae set also generated many features in the CSLB set and that the overall pattern of distribution of distinctive properties is similar.

### Comparison of feature correlations

McRae et al. (2005) measured feature correlation with intercorrelational density. This is the sum of squared correlations of all highly correlated (>6.5 % shared variance) property pairs within a concept. Tyler and colleagues (see Taylor et al., 2012, for a rationale) used a slightly different measure, mean correlational strength, which is the mean of the correlations of all significantly correlated property pairs within a concept. We calculated both of these measures for the 415 items in common between the two sets of norms, as well as the number of significantly correlated property pairs (CPPs) and the percentage of possible CPPs that are significantly correlated. Table 3 shows that all measures are highly correlated.

**Table 3:** Intercorrelational density (ICD), mean correlational strength (Corrstr), number of significantly correlated property pairs (CPPs), and percentage of feature pairs that are significantly correlated (%CPPs) for the 415 items in the McRae and CSLB norms, and their respective correlations across concepts

		ICD	Corrstr	CPPs	%CPPs
McRae	Mean	177	.26	8	23
	*SD*	208	.08	9	17
CSLB	Mean	298	.25	15	24
	*SD*	246	0.06	11	13
Correlation		.61	.36	.58	.35

We also calculated the mean correlational strength of each shared feature with all other features in the norms (using only significant CPPs). The CSLB norms have 598 features that are produced 3 or more times, and the McRae norms have 446. There were 341 shared features that were the same in both sets. The correlational strengths of these features are also highly correlated, *r* = .512, *p* < .001. This again indicates that participants produced very similar sets of features.

### Category structure and semantic similarity

**Fig. 2:**
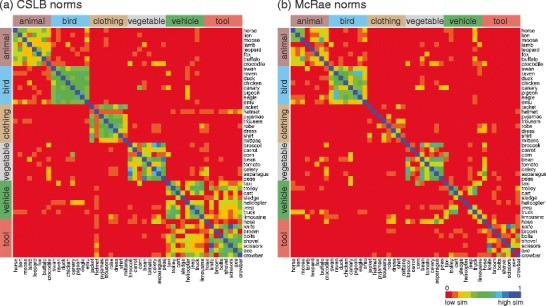
Similarity structure for 48 items in common for CSLB and McRae norms

**Fig. 3:**
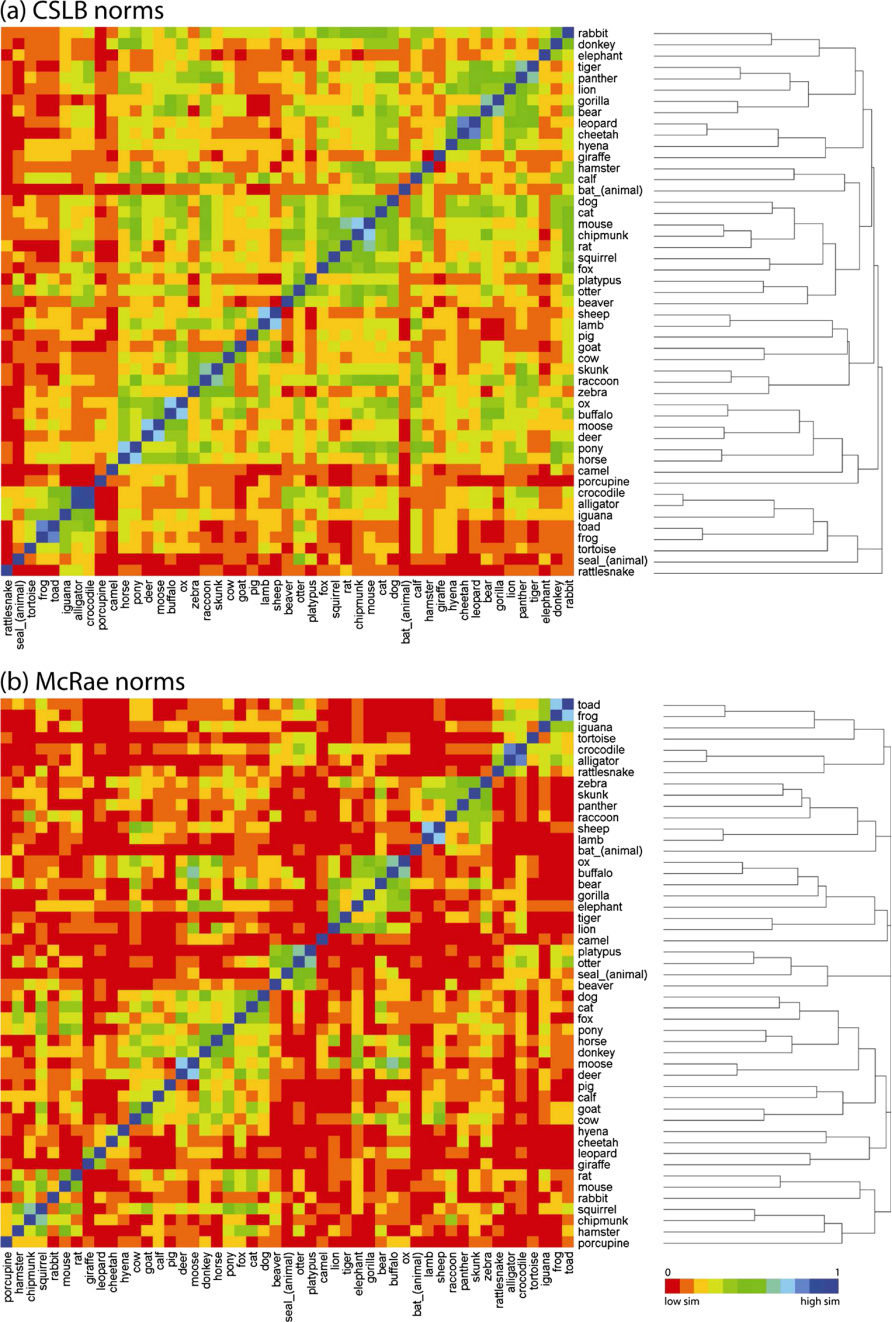
Similarity structure for the 49 “land animal” items appearing in both the CLSB and McRae norms. Rows and columns of the similarity matrix are ordered by a complete-linkage hierarchical clustering solution

One important aspect of feature-based accounts of conceptual representation is that shared information forms the basis of category organization. A category is formed when a set of features reliably co-occurs over a number of concepts. For example, we can tell that a swan is from the category of birds because it possesses the shared, correlated features of birds (*has wings*, *has feathers*, *lays eggs*, *flies*, *does nest*). A measure of the effectiveness of the norms that we have produced is the extent to which concepts from the same category are similar. The similarity of two concepts was calculated as the cosine between their two production frequency vectors (McRae et al., 2005). We calculated similarity matrices and hierarchical clustering solutions (excluding taxonomic features) and compared these for the McRae and CSLB norms, again using the production frequency cutoff of 5. The second-order similarity (i.e., correlation between pairwise cosine similarity matrices) between the McRae norms and the CSLB norms is highly significant (Spearman rho; for all 415 items, rho = .624, *p* < .0001). Figures 2 and 3 illustrate the similarity structure. For clarity, rather than depict the full similarity structure for 415 items, we show the similarity structure for 48 items (6 items drawn at random from the eight most common categories; Fig. 2).

**Fig. 4:**
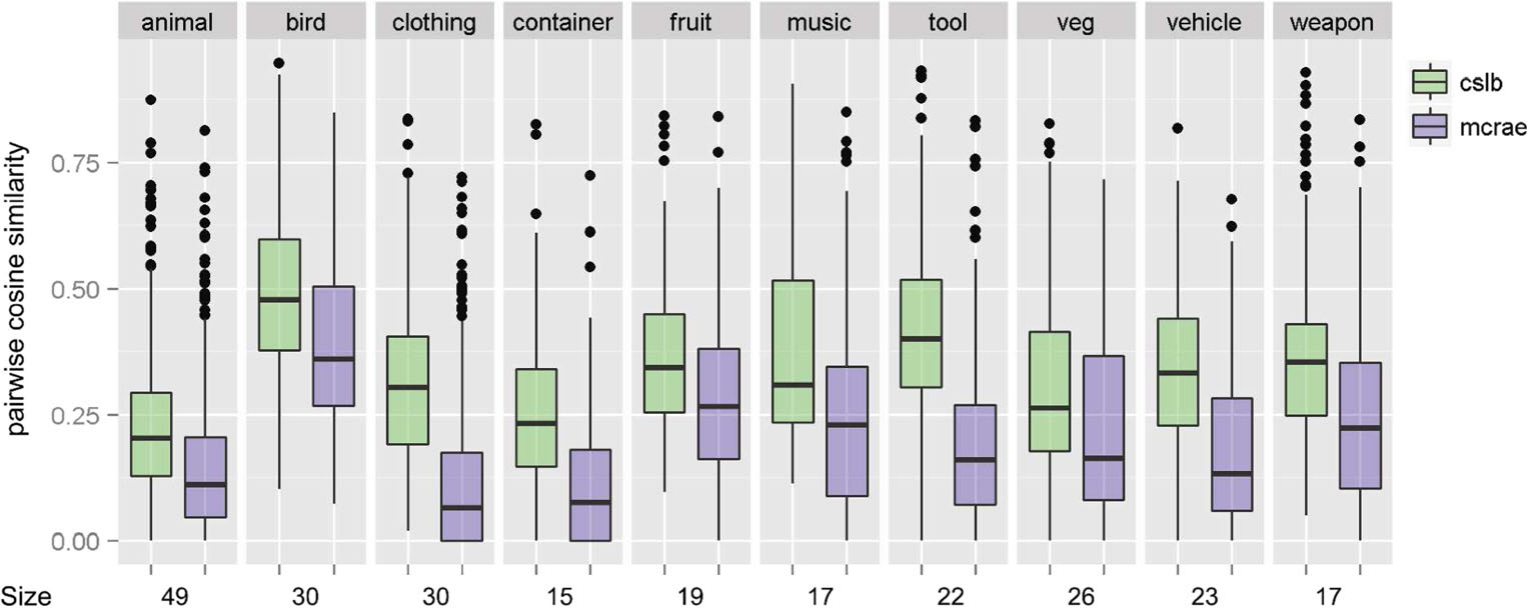
Within-category similarity for the McRae and CSLB norms. *Size*: the number of items common to both sets of norms in each category

The CSLB matrix has more intracategory pairs with high similarity (cooler colors) than the McRae matrix, indicating a tighter category structure (see also Fig. 4). We also report the full similarity structure and hierarchical clustering solution for all 49 items from the most common category (“land animals”). Here, there is clear organization by semantic similarity, for both the McRae and CLSB norms, with highly similar pairs of items (moose and buffalo; sheep and lamb) clustering together. In particular, for both sets of norms, amphibians and reptiles separate from the other animals. Similarity within the animal category is higher for the CSLB norms than for the McRae norms (cooler colors). More generally, Fig. 4 presents the average within-category similarity for the 10 largest semantic categories that we identified. For every category, within-category similarity is higher for the CSLB norms than for the McRae norms (all *p*s < .001, Wilcoxon signed-rank test on pairwise similarity values, Bonferroni-corrected for the number of categories tested). This tighter category structure may have arisen because we ensured, as far as possible, that all concepts had close semantic relatives in the set and through the automatic collation of features, which helps to ensure that synonyms are identified and, so, fewer spurious distinctive features remain.

## Conclusion

The number of large-scale property norms available for use in research into conceptual knowledge remains small, and there is an increasing awareness of the need to provide more information in the data sets (Buchanan et al., 2013). We offer here a new set of 638 norms and show that these norms have a large degree of overlap with the norms of McRae et al. (2005), which currently provide the gold standard for norm production.

Using the methods described here, we have developed a semiautomated pipeline for the collection and collation of large sets of norms. Our norms are able to offer a wider range of information than is currently available in other sets of norms. As well as the production frequency, researchers have access to the linguistic variations that have been collapsed to create any given normalized feature. Variations in the syntax of a feature, as well as synonyms, are available. This gives researchers the opportunity to manipulate and modify the norms, further collapsing or separating features depending on the requirements of a particular research goal. We anticipate that these norms will be particularly useful for researchers who wish to use the variations that people provide when listing features to train computational systems to create automatically generated norms.

The scope of these norms has the potential to support researchers in developing more detailed models of conceptual knowledge and their underlying representations.

## Electronic supplementary material

Below is the link to the electronic supplementary material.

ESM 1 (DOCX 47 kb)
