# Supplementary material for: The Centre for Speech, Language and the Brain (CSLB) concept property norms
Source: Behav Res Methods. 2013 Dec 20;46(4):1119–27. doi: 10.3758/s13428-013-0420-4 (PMC4237904; doi:10.3758/s13428-013-0420-4)
Supplement: Supplementary file 1 — (DOCX 47 kb) [file 13428_2013_420_MOESM1_ESM.docx]

**Appendix**

| **Num** | **Category** | **Concept** | **In McRae** | **McRae label** |
| --- | --- | --- | --- | --- |
| 1 | appliance | blender | yes | blender |
| 2 | appliance | camera | no |  |
| 3 | appliance | coffee machine | no |  |
| 4 | appliance | coffee pot | no |  |
| 5 | appliance | dishwasher | yes | dishwasher |
| 6 | appliance | fridge | yes | fridge |
| 7 | appliance | kettle | yes | kettle |
| 8 | appliance | kitchen scales | no |  |
| 9 | appliance | microwave | yes | microwave |
| 10 | appliance | oven | yes | oven |
| 11 | appliance | radio | yes | radio |
| 12 | appliance | stove | yes | stove |
| 13 | appliance | television | no |  |
| 14 | appliance | toaster | yes | toaster |
| 15 | appliance | typewriter | yes | typewriter |
| 16 | appliance | washing machine | no |  |
| 17 | bird | budgie | yes | budgie |
| 18 | bird | buzzard | yes | buzzard |
| 19 | bird | canary | yes | canary |
| 20 | bird | chicken | yes | chicken |
| 21 | bird | cockerel | yes | rooster |
| 22 | bird | crane (bird) | no |  |
| 23 | bird | dove | yes | dove |
| 24 | bird | duck | yes | duck |
| 25 | bird | eagle | yes | eagle |
| 26 | bird | emu | yes | emu |
| 27 | bird | falcon | yes | falcon |
| 28 | bird | flamingo | yes | flamingo |
| 29 | bird | goose | yes | goose |
| 30 | bird | hawk | yes | hawk |
| 31 | bird | heron | no |  |
| 32 | bird | hummingbird | no |  |
| 33 | bird | kingfisher | no |  |
| 34 | bird | magpie | no |  |
| 35 | bird | nightingale | yes | nightingale |
| 36 | bird | ostrich | yes | ostrich |
| 37 | bird | owl | yes | owl |
| 38 | bird | parakeet | yes | parakeet |
| 39 | bird | partridge | yes | partridge |
| 40 | bird | peacock | yes | peacock |
| 41 | bird | pelican | yes | pelican |
| 42 | bird | penguin | yes | penguin |
| 43 | bird | pigeon | yes | pigeon |
| 44 | bird | raven | yes | raven |
| 45 | bird | robin | yes | robin |
| 46 | bird | seagull | yes | seagull |
| 47 | bird | sparrow | yes | sparrow |
| 48 | bird | starling | yes | starling |
| 49 | bird | swan | yes | swan |
| 50 | bird | turkey | yes | turkey |
| 51 | bird | woodpecker | yes | woodpecker |
| 52 | bird | wren | no |  |
| 53 | body part | arm | no |  |
| 54 | body part | bone | no |  |
| 55 | body part | ear | no |  |
| 56 | body part | eye | no |  |
| 57 | body part | finger | no |  |
| 58 | body part | foot | no |  |
| 59 | body part | hair | no |  |
| 60 | body part | heart | no |  |
| 61 | body part | leg | no |  |
| 62 | body part | lips | no |  |
| 63 | body part | nose | no |  |
| 64 | body part | thumb | no |  |
| 65 | body part | toe | no |  |
| 66 | body part | tongue | no |  |
| 67 | building | bridge | yes | bridge |
| 68 | building | castle | no |  |
| 69 | building | pyramid | yes | pyramid |
| 70 | clothing | apron | yes | apron |
| 71 | clothing | armour | yes | armour |
| 72 | clothing | belt | yes | belt |
| 73 | clothing | bikini | no |  |
| 74 | clothing | blouse | yes | blouse |
| 75 | clothing | bra | yes | bra |
| 76 | clothing | cap | yes | cap (hat) |
| 77 | clothing | cape | yes | cape |
| 78 | clothing | cloak | yes | cloak |
| 79 | clothing | coat | yes | coat |
| 80 | clothing | dress | yes | dress |
| 81 | clothing | dressing gown | no |  |
| 82 | clothing | earmuffs | yes | earmuffs |
| 83 | clothing | gloves | yes | gloves |
| 84 | clothing | gown | yes | gown |
| 85 | clothing | helmet | yes | helmet |
| 86 | clothing | jacket | yes | jacket |
| 87 | clothing | jeans | yes | jeans |
| 88 | clothing | knickers | no |  |
| 89 | clothing | leotard | yes | leotards |
| 90 | clothing | mittens | yes | mittens |
| 91 | clothing | parka | yes | parka |
| 92 | clothing | pyjamas | yes | pajamas |
| 93 | clothing | robe | yes | robe |
| 94 | clothing | scarf | yes | scarf |
| 95 | clothing | shawl | yes | shawl |
| 96 | clothing | shirt | yes | shirt |
| 97 | clothing | skirt | yes | skirt |
| 98 | clothing | sock | yes | socks |
| 99 | clothing | stockings | no |  |
| 100 | clothing | suit | no |  |
| 101 | clothing | sweater | yes | sweater |
| 102 | clothing | swimsuit | yes | swimsuit |
| 103 | clothing | tights | no |  |
| 104 | clothing | trousers | yes | trousers |
| 105 | clothing | veil | yes | veil |
| 106 | clothing | wetsuit | no |  |
| 107 | container | ashtray | yes | ashtray |
| 108 | container | bag | yes | bag |
| 109 | container | barrel | yes | barrel |
| 110 | container | basin | no |  |
| 111 | container | basket | yes | basket |
| 112 | container | bath | yes | bathtub |
| 113 | container | bin | yes | bin (waste) |
| 114 | container | bottle | yes | bottle |
| 115 | container | bowl | yes | bowl |
| 116 | container | box | yes | box |
| 117 | container | bucket | yes | bucket |
| 118 | container | cage | yes | cage |
| 119 | container | cup | yes | cup |
| 120 | container | hutch | no |  |
| 121 | container | jar | yes | jar |
| 122 | container | jug | no |  |
| 123 | container | mug | yes | mug |
| 124 | container | satchel | no |  |
| 125 | container | sink | yes | sink |
| 126 | container | wallet | no |  |
| 127 | drink | beer | no |  |
| 128 | drink | brandy | no |  |
| 129 | drink | champagne | no |  |
| 130 | drink | cider | no |  |
| 131 | drink | coffee | no |  |
| 132 | drink | gin | no |  |
| 133 | drink | lemonade | no |  |
| 134 | drink | tea | no |  |
| 135 | drink | whisky | no |  |
| 136 | drink | wine | no |  |
| 137 | drug & cosmetic | aspirin | no |  |
| 138 | drug & cosmetic | codeine | no |  |
| 139 | drug & cosmetic | ibuprofen | no |  |
| 140 | drug & cosmetic | lipstick | no |  |
| 141 | drug & cosmetic | nail polish | no |  |
| 142 | drug & cosmetic | penicillin | no |  |
| 143 | drug & cosmetic | perfume | no |  |
| 144 | drug & cosmetic | valium | no |  |
| 145 | fish | carp | no |  |
| 146 | fish | cod | yes | cod |
| 147 | fish | eel | yes | eel |
| 148 | fish | flounder | no |  |
| 149 | fish | goldfish | yes | goldfish |
| 150 | fish | herring | no |  |
| 151 | fish | mackerel | yes | mackerel |
| 152 | fish | minnow | yes | minnow |
| 153 | fish | salmon | yes | salmon |
| 154 | fish | sardine | yes | sardine |
| 155 | fish | seahorse | no |  |
| 156 | fish | shark | no |  |
| 157 | fish | trout | yes | trout |
| 158 | fish | tuna | yes |  |
| 159 | flower | buttercup | no |  |
| 160 | flower | carnation | no |  |
| 161 | flower | daffodil | no |  |
| 162 | flower | daisy | no |  |
| 163 | flower | dandelion | yes | dandelion |
| 164 | flower | geranium | no |  |
| 165 | flower | hyacinth | no |  |
| 166 | flower | lily | no |  |
| 167 | flower | marigold | no |  |
| 168 | flower | orchid | no |  |
| 169 | flower | pansy | no |  |
| 170 | flower | poppy | no |  |
| 171 | flower | rose | no |  |
| 172 | flower | sunflower | no |  |
| 173 | flower | tulip | no |  |
| 174 | food | bacon | no |  |
| 175 | food | biscuit | yes | biscuit |
| 176 | food | bread | yes | bread |
| 177 | food | butter | no |  |
| 178 | food | cake | yes | cake |
| 179 | food | cheese | yes | cheese |
| 180 | food | chocolate | no |  |
| 181 | food | currant | no |  |
| 182 | food | doughnut | no |  |
| 183 | food | flour | no |  |
| 184 | food | gherkin | yes | pickle |
| 185 | food | ham | no |  |
| 186 | food | ice cream | no |  |
| 187 | food | jam | no |  |
| 188 | food | jelly | no |  |
| 189 | food | ketchup | no |  |
| 190 | food | mayonnaise | no |  |
| 191 | food | milk | no |  |
| 192 | food | pie | yes | pie |
| 193 | food | prune | yes | prune |
| 194 | food | raisin | yes | raisin |
| 195 | food | rice | yes | rice |
| 196 | food | sandwich | no |  |
| 197 | food | soup | no |  |
| 198 | food | sugar | no |  |
| 199 | food | sultana | no |  |
| 200 | food | yoghurt | no |  |
| 201 | footwear | boots | yes | boots |
| 202 | footwear | flip flops | no |  |
| 203 | footwear | sandals | yes | sandals |
| 204 | footwear | shoes | yes | shoes |
| 205 | footwear | slippers | yes | slippers |
| 206 | fruit | apple | yes | apple |
| 207 | fruit | apricot | no |  |
| 208 | fruit | banana | yes | banana |
| 209 | fruit | blueberry | yes | blueberry |
| 210 | fruit | cherry | yes | cherry |
| 211 | fruit | coconut | yes | coconut |
| 212 | fruit | dates (fruit) | no |  |
| 213 | fruit | grape | yes | grape |
| 214 | fruit | grapefruit | yes | grapefruit |
| 215 | fruit | kiwi fruit | no |  |
| 216 | fruit | lemon | yes | lemon |
| 217 | fruit | lime | yes | lime |
| 218 | fruit | mango | no |  |
| 219 | fruit | melon | no |  |
| 220 | fruit | nectarine | yes | nectarine |
| 221 | fruit | nut | no |  |
| 222 | fruit | orange | yes | orange |
| 223 | fruit | peach | yes | peach |
| 224 | fruit | peanut | no |  |
| 225 | fruit | pear | yes | pear |
| 226 | fruit | pineapple | yes | pineapple |
| 227 | fruit | plum | yes | plum |
| 228 | fruit | raspberry | yes | raspberry |
| 229 | fruit | rhubarb | yes | rhubarb |
| 230 | fruit | satsuma | no |  |
| 231 | fruit | strawberry | yes | strawberry |
| 232 | fruit | tangerine | yes | tangerine |
| 233 | fruit | watermelon | no |  |
| 234 | furniture | armchair | no |  |
| 235 | furniture | bed | yes | bed |
| 236 | furniture | bench | yes | bench |
| 237 | furniture | bookcase | yes | bookcase |
| 238 | furniture | chair | yes | chair |
| 239 | furniture | chandelier | yes | chandelier |
| 240 | furniture | chest of drawers | no |  |
| 241 | furniture | clock | yes | clock |
| 242 | furniture | cradle | no |  |
| 243 | furniture | cupboard | yes | cupboard |
| 244 | furniture | deckchair | no |  |
| 245 | furniture | desk | yes | desk |
| 246 | furniture | dresser | yes | dresser |
| 247 | furniture | lamp | yes | lamp |
| 248 | furniture | mirror | yes | mirror |
| 249 | furniture | rocking chair | yes | rocker |
| 250 | furniture | sofa | yes | sofa |
| 251 | furniture | stool | yes | stool (furniture) |
| 252 | furniture | table | yes | table |
| 253 | furniture | throne | no |  |
| 254 | invertebrate | ant | yes | ant |
| 255 | invertebrate | bee | no |  |
| 256 | invertebrate | beetle | yes | beetle |
| 257 | invertebrate | butterfly | yes | butterfly |
| 258 | invertebrate | caterpillar | yes | caterpillar |
| 259 | invertebrate | centipede | no |  |
| 260 | invertebrate | cockroach | yes | cockroach |
| 261 | invertebrate | cricket | no |  |
| 262 | invertebrate | dragonfly | no |  |
| 263 | invertebrate | flea | yes | flea |
| 264 | invertebrate | grasshopper | yes | grasshopper |
| 265 | invertebrate | hornet | yes | hornet |
| 266 | invertebrate | housefly | yes | housefly |
| 267 | invertebrate | ladybird | no |  |
| 268 | invertebrate | locust | no |  |
| 269 | invertebrate | millipede | no |  |
| 270 | invertebrate | mosquito | no |  |
| 271 | invertebrate | moth | yes | moth |
| 272 | invertebrate | scorpion | no |  |
| 273 | invertebrate | slug | no |  |
| 274 | invertebrate | snail | yes | snail |
| 275 | invertebrate | spider | yes | spider |
| 276 | invertebrate | stick insect | no |  |
| 277 | invertebrate | tarantula | no |  |
| 278 | invertebrate | termite | no |  |
| 279 | invertebrate | wasp | yes | wasp |
| 280 | invertebrate | worm | yes | worm |
| 281 | jewellery | bracelet | yes | bracelet |
| 282 | jewellery | necklace | yes | necklace |
| 283 | jewellery | ring (jewellery) | yes | ring (jewelry) |
| 284 | kitchenware | can opener | no |  |
| 285 | kitchenware | colander | yes | colander |
| 286 | kitchenware | corkscrew | yes | corkscrew |
| 287 | kitchenware | cutting board | yes | board (wood) |
| 288 | kitchenware | fork | yes | fork |
| 289 | kitchenware | grater | yes | grater |
| 290 | kitchenware | ladle | yes | ladle |
| 291 | kitchenware | peeler | no |  |
| 292 | kitchenware | plate | yes | plate |
| 293 | kitchenware | rolling pin | no |  |
| 294 | kitchenware | spatula | yes | spatula |
| 295 | kitchenware | spoon | yes | spoon |
| 296 | kitchenware | strainer | yes | strainer |
| 297 | kitchenware | tray | yes | tray |
| 298 | kitchenware | whisk | no |  |
| 299 | land animal | alligator | yes | alligator |
| 300 | land animal | bat (animal) | yes | bat (animal) |
| 301 | land animal | bear | yes | bear |
| 302 | land animal | beaver | yes | beaver |
| 303 | land animal | buffalo | yes | buffalo |
| 304 | land animal | calf | yes | calf |
| 305 | land animal | camel | yes | camel |
| 306 | land animal | cat | yes | cat |
| 307 | land animal | cheetah | yes | cheetah |
| 308 | land animal | chipmunk | yes | chipmunk |
| 309 | land animal | cow | yes | cow |
| 310 | land animal | crocodile | yes | crocodile |
| 311 | land animal | deer | yes | deer |
| 312 | land animal | dog | yes | dog |
| 313 | land animal | donkey | yes | donkey |
| 314 | land animal | elephant | yes | elephant |
| 315 | land animal | fox | yes | fox |
| 316 | land animal | frog | yes | frog |
| 317 | land animal | gerbil | no |  |
| 318 | land animal | giraffe | yes | giraffe |
| 319 | land animal | goat | yes | goat |
| 320 | land animal | gorilla | yes | gorilla |
| 321 | land animal | guinea pig | no |  |
| 322 | land animal | hamster | yes | hamster |
| 323 | land animal | hedgehog | no |  |
| 324 | land animal | hippo | no |  |
| 325 | land animal | horse | yes | horse |
| 326 | land animal | hyena | yes | hyena |
| 327 | land animal | iguana | yes | iguana |
| 328 | land animal | kangaroo | no |  |
| 329 | land animal | lamb | yes | lamb |
| 330 | land animal | leopard | yes | leopard |
| 331 | land animal | lion | yes | lion |
| 332 | land animal | lizard | no |  |
| 333 | land animal | llama | no |  |
| 334 | land animal | monkey | no |  |
| 335 | land animal | moose | yes | moose |
| 336 | land animal | mouse | yes | mouse |
| 337 | land animal | otter | yes | otter |
| 338 | land animal | ox | yes | ox |
| 339 | land animal | panther | yes | panther |
| 340 | land animal | pig | yes | pig |
| 341 | land animal | platypus | yes | platypus |
| 342 | land animal | pony | yes | pony |
| 343 | land animal | porcupine | yes | porcupine |
| 344 | land animal | rabbit | yes | rabbit |
| 345 | land animal | raccoon | yes | raccoon |
| 346 | land animal | rat | yes | rat |
| 347 | land animal | rattlesnake | yes | rattlesnake |
| 348 | land animal | rhino | no |  |
| 349 | land animal | seal | yes | seal |
| 350 | land animal | sheep | yes | sheep |
| 351 | land animal | skunk | yes | skunk |
| 352 | land animal | squirrel | yes | squirrel |
| 353 | land animal | tiger | yes | tiger |
| 354 | land animal | toad | yes | toad |
| 355 | land animal | tortoise | yes | tortoise |
| 356 | land animal | wolf | no |  |
| 357 | land animal | zebra | yes | zebra |
| 358 | misc | anchor | yes | anchor |
| 359 | misc | badge | no |  |
| 360 | misc | bat (sporting) | yes | bat (baseball) |
| 361 | misc | bell | no |  |
| 362 | misc | bellows | no |  |
| 363 | misc | bouquet | yes | bouquet |
| 364 | misc | brick | yes | brick |
| 365 | misc | brush | yes | brush |
| 366 | misc | buckle | yes | buckle |
| 367 | misc | button | no |  |
| 368 | misc | candle | yes | candle |
| 369 | misc | certificate | yes | certificate |
| 370 | misc | chain | yes | chain |
| 371 | misc | cigar | yes | cigar |
| 372 | misc | cigarette | yes | cigarette |
| 373 | misc | coffin | no |  |
| 374 | misc | coin | yes | coin |
| 375 | misc | comb | yes | comb |
| 376 | misc | cork | yes | cork |
| 377 | misc | crane (equipment) | yes | crane (machine) |
| 378 | misc | crayon | yes | crayon |
| 379 | misc | cushion | yes | cushion |
| 380 | misc | doorknob | yes | doorknob |
| 381 | misc | envelope | yes | envelope |
| 382 | misc | fence | yes | fence |
| 383 | misc | fern | no |  |
| 384 | misc | flannel | no |  |
| 385 | misc | gate | yes | gate |
| 386 | misc | glue | no |  |
| 387 | misc | goggles | no |  |
| 388 | misc | greeting card | yes | card (greeting) |
| 389 | misc | hook | yes | hook |
| 390 | misc | key | yes | key |
| 391 | misc | ladder | no |  |
| 392 | misc | lantern | yes | lantern |
| 393 | misc | mask | no |  |
| 394 | misc | mat | yes | mat |
| 395 | misc | microscope | yes | microscope |
| 396 | misc | mop | no |  |
| 397 | misc | moss | no |  |
| 398 | misc | napkin | yes | napkin |
| 399 | misc | needle | no |  |
| 400 | misc | peg | yes | peg |
| 401 | misc | pen | yes | pen |
| 402 | misc | pencil | yes | pencil |
| 403 | misc | pillow | yes | pillow |
| 404 | misc | pin | yes | pin |
| 405 | misc | pipe (smoking) | yes | pipe (smoking) |
| 406 | misc | poison | no |  |
| 407 | misc | pram | no |  |
| 408 | misc | projector | yes | projector |
| 409 | misc | razor | yes | razor |
| 410 | misc | rock | yes | rock |
| 411 | misc | rope | yes | rope |
| 412 | misc | ruler | yes | ruler |
| 413 | misc | sandpaper | yes | sandpaper |
| 414 | misc | seashell | yes | shell |
| 415 | misc | seaweed | yes | seaweed |
| 416 | misc | sellotape | yes | tape (scotch) |
| 417 | misc | shield | yes | shield |
| 418 | misc | skis | yes | skis |
| 419 | misc | stick | yes | stick |
| 420 | misc | stone | yes | stone |
| 421 | misc | surfboard | yes | surfboard |
| 422 | misc | syringe | no |  |
| 423 | misc | telephone | yes | telephone |
| 424 | misc | tent | yes | tent |
| 425 | misc | thermometer | yes | thermometer |
| 426 | misc | thimble | yes | thimble |
| 427 | misc | tobacco | no |  |
| 428 | misc | toilet | yes | toilet |
| 429 | misc | tongs | yes | tongs |
| 430 | misc | tripod | yes | tripod |
| 431 | misc | tyre | no |  |
| 432 | misc | umbrella | yes | umbrella |
| 433 | misc | wand | yes | wand |
| 434 | misc | watch | no |  |
| 435 | misc | wheel | yes | wheel |
| 436 | misc | whip | yes | whip |
| 437 | misc | whistle | yes | whistle |
| 438 | misc | window | no |  |
| 439 | music | accordion | yes | accordion |
| 440 | music | bagpipes | yes | bagpipe |
| 441 | music | banjo | yes | banjo |
| 442 | music | castanets | no |  |
| 443 | music | cello | yes | cello |
| 444 | music | clarinet | yes | clarinet |
| 445 | music | cymbal | no |  |
| 446 | music | drum | yes | drum |
| 447 | music | flute | yes | flute |
| 448 | music | french horn | no |  |
| 449 | music | gong | no |  |
| 450 | music | guitar | yes | guitar |
| 451 | music | harmonica | yes | harmonica |
| 452 | music | harp | yes | harp |
| 453 | music | harpsichord | yes | harpsichord |
| 454 | music | organ | no |  |
| 455 | music | piano | yes | piano |
| 456 | music | recorder | no |  |
| 457 | music | saxophone | yes | saxophone |
| 458 | music | tambourine | no |  |
| 459 | music | trombone | yes | trombone |
| 460 | music | trumpet | yes | trumpet |
| 461 | music | tuba | yes | tuba |
| 462 | music | violin | yes | violin |
| 463 | reading | book | yes | book |
| 464 | reading | brochure | no |  |
| 465 | reading | catalogue | no |  |
| 466 | reading | dictionary | no |  |
| 467 | reading | encyclopaedia | no |  |
| 468 | reading | menu | yes | menu |
| 469 | reading | newspaper | no |  |
| 470 | reading | pamphlet | no |  |
| 471 | reading | textbook | no |  |
| 472 | sea creature | clam | yes | clam |
| 473 | sea creature | crab | yes | crab |
| 474 | sea creature | crayfish | no |  |
| 475 | sea creature | dolphin | yes | dolphin |
| 476 | sea creature | jellyfish | no |  |
| 477 | sea creature | lobster | yes | lobster |
| 478 | sea creature | mussel | no |  |
| 479 | sea creature | octopus | yes | octopus |
| 480 | sea creature | oyster | no |  |
| 481 | sea creature | prawn | no |  |
| 482 | sea creature | scallop | no |  |
| 483 | sea creature | shrimp | yes | shrimp |
| 484 | sea creature | squid | yes | squid |
| 485 | sea creature | turtle | yes | turtle |
| 486 | sea creature | walrus | yes | walrus |
| 487 | sea creature | whale | yes | whale |
| 488 | tool | axe | yes | axe |
| 489 | tool | bolts | yes | bolts |
| 490 | tool | broom | yes | broom |
| 491 | tool | chainsaw | no |  |
| 492 | tool | chisel | yes | chisel |
| 493 | tool | clamp | yes | clamp |
| 494 | tool | crowbar | yes | crowbar |
| 495 | tool | drill | yes | drill |
| 496 | tool | hammer | yes | hammer |
| 497 | tool | hatchet | yes | hatchet |
| 498 | tool | hoe | yes | hoe |
| 499 | tool | hose | yes | hose |
| 500 | tool | knife | yes | knife |
| 501 | tool | machete | yes | machete |
| 502 | tool | nail (tool) | no |  |
| 503 | tool | penknife | no |  |
| 504 | tool | pliers | yes | pliers |
| 505 | tool | plough | no |  |
| 506 | tool | rake | yes | rake |
| 507 | tool | saw | no |  |
| 508 | tool | scalpel | no |  |
| 509 | tool | scissors | yes | scissors |
| 510 | tool | screw | yes | screws |
| 511 | tool | screwdriver | yes | screwdriver |
| 512 | tool | scythe | no |  |
| 513 | tool | shovel | yes | shovel |
| 514 | tool | spade | yes | spade |
| 515 | tool | spanner | yes | wrench |
| 516 | tool | spirit level | yes | level |
| 517 | tool | trowel | no |  |
| 518 | tool | tweezers | no |  |
| 519 | toy | ball | yes | ball |
| 520 | toy | balloon | yes | balloon |
| 521 | toy | boomerang | no |  |
| 522 | toy | dice | no |  |
| 523 | toy | doll | yes | doll |
| 524 | toy | football | yes | football |
| 525 | toy | frisbee | no |  |
| 526 | toy | kite | yes | kite |
| 527 | toy | marble | yes | marble |
| 528 | toy | puppet | no |  |
| 529 | toy | rattle | yes | rattle |
| 530 | toy | rollerskate | no |  |
| 531 | toy | skateboard | yes | skateboard |
| 532 | toy | swing | no |  |
| 533 | toy | yo-yo | no |  |
| 534 | tree | birch | yes | birch |
| 535 | tree | chestnut | no |  |
| 536 | tree | elm | no |  |
| 537 | tree | eucalyptus | no |  |
| 538 | tree | fir | no |  |
| 539 | tree | oak | yes | oak |
| 540 | tree | pine | yes | pine |
| 541 | tree | sycamore | no |  |
| 542 | tree | willow | yes | willow |
| 543 | vegetable | artichoke | no |  |
| 544 | vegetable | asparagus | yes | asparagus |
| 545 | vegetable | aubergine | yes | eggplant |
| 546 | vegetable | avocado | yes | avocado |
| 547 | vegetable | bean | yes | beans |
| 548 | vegetable | broccoli | yes | broccoli |
| 549 | vegetable | brussel sprouts | no |  |
| 550 | vegetable | cabbage | yes | cabbage |
| 551 | vegetable | carrot | yes | carrot |
| 552 | vegetable | cauliflower | yes | cauliflower |
| 553 | vegetable | celery | yes | celery |
| 554 | vegetable | corn | yes | corn |
| 555 | vegetable | courgette | yes | zucchini |
| 556 | vegetable | cucumber | yes | cucumber |
| 557 | vegetable | garlic | yes | garlic |
| 558 | vegetable | leek | no |  |
| 559 | vegetable | lettuce | yes | lettuce |
| 560 | vegetable | mushroom | yes | mushroom |
| 561 | vegetable | olive | yes | olive |
| 562 | vegetable | onion | yes | onions |
| 563 | vegetable | parsley | yes | parsley |
| 564 | vegetable | peas | yes | peas |
| 565 | vegetable | potato | yes | potato |
| 566 | vegetable | pumpkin | yes | pumpkin |
| 567 | vegetable | radish | yes | radish |
| 568 | vegetable | spinach | yes | spinach |
| 569 | vegetable | sweet potato | yes | yam |
| 570 | vegetable | tomato | yes | tomato |
| 571 | vegetable | turnip | yes | turnip |
| 572 | vehicle | aeroplane | yes | airplane |
| 573 | vehicle | ambulance | yes | ambulance |
| 574 | vehicle | bicycle | yes | bike |
| 575 | vehicle | buggy | yes | buggy |
| 576 | vehicle | bus | yes | bus |
| 577 | vehicle | car | yes | car |
| 578 | vehicle | caravan | no |  |
| 579 | vehicle | carriage | no |  |
| 580 | vehicle | cart | yes | cart |
| 581 | vehicle | coach | no |  |
| 582 | vehicle | glider | no |  |
| 583 | vehicle | helicopter | yes | helicopter |
| 584 | vehicle | jeep | yes | jeep |
| 585 | vehicle | land rover | no |  |
| 586 | vehicle | limousine | yes | limousine |
| 587 | vehicle | lorry | no |  |
| 588 | vehicle | moped | no |  |
| 589 | vehicle | motorcycle | yes | motorcycle |
| 590 | vehicle | porsche | no |  |
| 591 | vehicle | range rover | no |  |
| 592 | vehicle | rocket | yes | rocket |
| 593 | vehicle | rolls royce | no |  |
| 594 | vehicle | sledge | yes | sleigh |
| 595 | vehicle | tank | yes | tank (army) |
| 596 | vehicle | taxi | yes | taxi |
| 597 | vehicle | tractor | yes | tractor |
| 598 | vehicle | train | yes | train |
| 599 | vehicle | tricycle | yes | tricycle |
| 600 | vehicle | trolley | yes | trolley |
| 601 | vehicle | truck | yes | truck |
| 602 | vehicle | unicycle | yes | unicycle |
| 603 | vehicle | van | yes | van |
| 604 | vehicle | wheelbarrow | yes | wheelbarrow |
| 605 | vehicle | wheelchair | no |  |
| 606 | water vehicle | barge | no |  |
| 607 | water vehicle | boat | yes | boat |
| 608 | water vehicle | canoe | yes | canoe |
| 609 | water vehicle | dinghy | no |  |
| 610 | water vehicle | ferry | no |  |
| 611 | water vehicle | houseboat | no |  |
| 612 | water vehicle | kayak | no |  |
| 613 | water vehicle | oil tanker | no |  |
| 614 | water vehicle | raft | yes | raft |
| 615 | water vehicle | ship | yes | ship |
| 616 | water vehicle | speedboat | no |  |
| 617 | water vehicle | submarine | yes | submarine |
| 618 | water vehicle | tugboat | no |  |
| 619 | water vehicle | yacht | yes | yacht |
| 620 | weapon | arrow | no |  |
| 621 | weapon | bayonet | yes | bayonet |
| 622 | weapon | bomb | yes | bomb |
| 623 | weapon | bow (weapon) | yes | bow (weapon) |
| 624 | weapon | bullet | yes | bullet |
| 625 | weapon | cannon | yes | cannon |
| 626 | weapon | catapult | yes | catapult |
| 627 | weapon | club | no |  |
| 628 | weapon | crossbow | yes | crossbow |
| 629 | weapon | dagger | yes | dagger |
| 630 | weapon | grenade | yes | grenade |
| 631 | weapon | gun | yes | gun |
| 632 | weapon | harpoon | yes | harpoon |
| 633 | weapon | pistol | yes | pistol |
| 634 | weapon | revolver | yes | revolver |
| 635 | weapon | rifle | yes | rifle |
| 636 | weapon | shotgun | yes | shotgun |
| 637 | weapon | spear | yes | spear |
| 638 | weapon | sword | yes | sword |
